# Some Scars Do Not Run Deep: The Qualitative and Quantitative Nature of Neonatal Seldinger Pigtail Chest Drain Scars

**DOI:** 10.1177/1179556519855384

**Published:** 2019-06-11

**Authors:** Leo BH Gundle, Aimee Dowek, Priya Heer, Steven Jones, David Bartle

**Affiliations:** 1 Bristol Medical School, University of Bristol, Bristol, UK; 2 Medical School, University of Exeter, Exeter, UK; 3 Neonatal Intensive Care Unit, Royal Devon and Exeter NHS Foundation Trust, Exeter, UK; 4 Neonatal Intensive Care Unit, Royal United Hospitals Bath, Bath, UK

**Keywords:** quality improvement, pulmonology or respiratory disorders, newborn

## Abstract

Pneumothorax is a complication of respiratory distress syndrome, of which many preterm babies suffer. If significant, these pneumothoraces can be treated by the insertion of a chest drain. There are a number of different types of chest drain, and techniques of insertion. This study aims to establish both the quantitative nature, and emotional significance of neonatal pigtail chest drain scarring, as inserted via the Seldinger technique. Parents were interviewed by telephone and asked to send photographs of their child’s scar to be graded. Researchers found that, on the whole, the scar size and severity was not significant, and that those interviewed generally agreed that while the scar served as a reminder of a traumatic time, its size was unimportant. These results may inform future practice on a basis of chest drain comparison; results imply that chest drains with the greatest efficacy should be used.

## Introduction

### Background

In 2012, over 52,000 babies were born prematurely in the United Kingdom, equating to 7% to 8% of all live births.^1^ Premature birth is defined as birth before 37 weeks of gestation and is associated with an increased risk of a number of post-birth complications due to foetal underdevelopment.^2,3^ Consequently, premature babies are cared for on a specialised neonatal intensive care unit (NICU).

Complications are influenced by many factors, one of which is being the extent of prematurity. For example, infants born before 34 weeks of gestation may lack the surfactant-producing alveolar cells necessary for lung structure stability and healthy respiration.^2^ As a consequence, they are at risk of developing respiratory distress syndrome (RDS).^2,3^

The management of RDS often involves the neonate being mechanically ventilated until surfactant has been administered and their oxygen saturations are satisfactory.^3^ Ventilation is an invasive treatment and occasionally can cause associated complications, eg, a pneumothorax.^4^

Pneumothoraces are caused by alveoli being overinflated and rupturing, causing air to leak in between the visceral and parietal lung pleura.^4,5^ If significant, it will be treated immediately through the use of a chest drain, commonly inserted using the Seldinger technique. This technique involves making a small incision in the fifth intercostal space, midaxillary line, where a small tube can be inserted into the chest cavity, allowing the air to be drained from the chest cavity and the lungs to re-inflate.^6^

The NICU at the Royal United Hospitals Bath (RUH) and Royal Devon and Exeter Hospital (RD&E) use pigtail drains, requiring a smaller incision than the alternative traditional chest drain.^7^ The scar from this procedure is in a discreet location: either the 5th intercostal space midaxillary line or mid-clavicular line in females, such that it may be hidden by the breast later in the child’s life. Nonetheless, it is a scar and will be present for a considerable period of the patient’s life, if not throughout.

Although there is great deal of research on the complications of pneumothoraces and chest drains, the emotional impact that the scars have on the parents, once the baby has left the neonatal unit, has not previously been evaluated. This novel investigation will give researchers an insight into the extent that chest drain scars have an emotional impact upon mothers, as well as quantitatively measuring the nature of scarring caused by the pigtail drain, as inserted via the Seldinger technique.

### Aim

To (1) quantify the pigtail chest drain scars and (2) investigate the emotional impact that neonatal chest drain scars have on mothers.

#### Objectives

Identify patients who had a chest drain in the NICU unit in the RUH and RD&E, between 2008 and 2018.
Contact and interview parents on the emotional impact of their child’s chest drain scars.
To gather photographs of the chest drain scars and analyse them using the Stony Brook Scar Evaluation Scale.
To collect data to compare with future research on alternative chest drains.
To evaluate whether the effects of the scar needs to be considered in practice.

## Method

### Patient identification

RUH and RD&E records on BadgerNet were analysed from February 2008 to January 2018. In total, 97 patients were identified as having had a pneumothorax. Discharge summaries of each case were then examined to establish whether the patient had received a subsequent chest drain. In total, 39 such patients were identified.

Of these 39 patients, records yielded parent telephone numbers for 31. Parents were all contacted at least two times for interview. After two cycles of contacting patients, successful interviews were held with nine, with six photos of patient scars being received. Reasons for loss of data for remaining numbers include old telephone numbers, lack of availability of parent, and unwillingness to speak to researchers.

### Interview

Verbal informed consent was obtained via telephone and receipt of the photography from the parents. Parents of patients who had received chest drains were interviewed over the phone. Researchers asked primarily open questions, to establish qualitatively how parents reacted to their child’s scar and to see whether any common themes arose. Questions were designed primarily such that researchers could understand the extent to which scars emotionally affect the parent or serve as a traumatic reminder. They were written by researchers in conjunction with senior colleagues from the NICU at the Royal United Hospital Bath. Specific ethics approval was not required, as researchers were undertaking a review of services.

The same interview questions were asked at each phone interview, see Table 1.

**Table 1.** Questions asked to parent.

Question number	Question asked to parent
1	Does the scar serve as a reminder of a traumatic or worrying time?
1a	If so, how?
2	Would it make a difference if the scar was smaller?
2a	Why or why not?
3	Does your child’s scar have an emotional impact on you?
3a	If so, how?

Parents were asked three closed questions during the telephone interview. Based on these answers, interviewers followed with an open sub-question, to further explore the parent’s answers.

### Data collection

#### Interview

Parents were interviewed over the phone. Interviews were anonymised, and key answers to questions were documented verbatim. From there, common themes which arose were collected and coded according to qualitative research techniques. Keywords (and close synonyms thereof) formed the basis of the researchers’ coding.

#### Photographs of scars

Parents were asked to take a photograph of their child’s scar and email it to researchers on National Health Service secure servers. Photos were anonymised and stored securely online, where they were analysed.

### Grading system

A number of scar scoring systems have been identified, which seek to quantify scarring. Those identified by researchers were The Vancouver Scar Scale, Patient and Observer Scar Assessment Scale, and The Stony Brook Scar Evaluation Scale.^8–10^ The Stony Brook Scar Evaluation Scale was deemed to be most appropriate to the photographic data; other systems required a closer examination of the scar tissue, and this was not available to researchers. The criteria of the Stony Brook Scar Evaluation Scale can be found in Table 2.

**Table 2.** Stony Brook Scar Evaluation Scale.^8^

	Scar category	Points
Width	>2 mm	0
	⩽2 mm	1
Height	Elevated/depressed in relation to surrounding skin	0
	Flat	1
Colour	Darker than surrounding skin	0
	Same colour or lighter than surrounding skin	1
Hatch marks/suture mars	Present	0
	Absent	1
Overall appearance	Poor	0
	Good	1

The Stony Brook Scar Evaluation Scale was considered to be most suitable for the data that researchers were collecting. The best possible score is 5, and the worst is 0. A score of 5 indicates a small and well-healed scar. The criteria for this scale are outlined in this table.

## Results

### Interview results

Nine parents were interviewed in this study.

During interviews, a number of common themes were identified by parents when answering questions provided by the interviewers.

#### Scar serving as an emotional trigger

An area of interest for researchers was the extent to which the sight of the scar can trigger a negative emotional response. This response would unnecessarily remind the parent of a worrisome time in the NICU.

When asked directly, just under half of those interviewed (4 out of 9) reported that the scar does act to provoke an emotional reminder of a traumatic time with their child in the NICU. One parent reported the scar as follows: ‘It reminds me [parent] of all the things he [the patient] has had to go through to still be here’.

Another parent reported that the scar acts as a constant reminder of the traumatic birth; the patient received a great deal of medical intervention, as well as a chest drain. The mother reports that the scar, and the experiences connected with it, is a permanent fixture on her mind. As a consequence, she suffers from post-traumatic stress disorder, with the scar being a significant intensifier of the memory of her experience.

Another mother reported that she connects the sight of the scar with the memory of being told that her ‘baby may not make it’. This was something that had a considerable emotional effect on the mother, and the scar serves as a reminder of that.

The remaining parents who were interviewed (5 out of 9), however reported that their child’s scar did not act as any significant emotional trigger. Three mothers when directly asked if the scar serves as an emotional reminder simply replied ‘no’, with no significant expansion or elaboration on their response when encouraged by researches.

A third mother reported that the memory of her child in the NICU was always present and with her, and that the scar did not act to intensify or specifically remind her of this. She went on to say ‘scar or no scar, the memory is always with you’.

#### Importance of scar size as a reminder

Researchers were interested in investigating if the size of a chest drain scar was important to parents. When directly asked, six out of the nine parents reported that the size of the scar would not make a difference. The general consensus among those reported scar size as being unimportant was that either the scar had an emotional impact on them or it did not, and that the size of the scar was irrelevant to this. One parent volunteered the following: ‘It wouldn’t matter if the scar was redder or angrier, it would remind me as much either way’.

Two parents reported to interviewers that the size of the scar would only matter if they were significantly larger. The implication to interviewers was that the scar size would have to be a number of times larger, and that a scar twice or three times as large would not be classed as significant to these parents. One of the nine parents felt that an increase in the scar size was an important factor in terms of its appearance and emotional impact.

#### Scars emotionally impacting parents

One of the aims for researchers was to identify the overall emotional importance of a scar, both as a trigger for a traumatic memory, but also specifically as an upsetting physical feature. When asked if the scar itself has an emotional impact on the parent, three said yes, with the remainder saying no. The impact however was in the context of the scar acting as a reminder of the baby’s traumatic birth, rather than as an upsetting feature in of itself.

A mother said to researchers that the scar ‘always reminds me [the mother] of the trauma of the birth’. The remainder of parents interviewed (6 out of 9) said that the scar itself was not emotionally upsetting, with four saying the scar was hardly noticeable.

**Figure 1.**
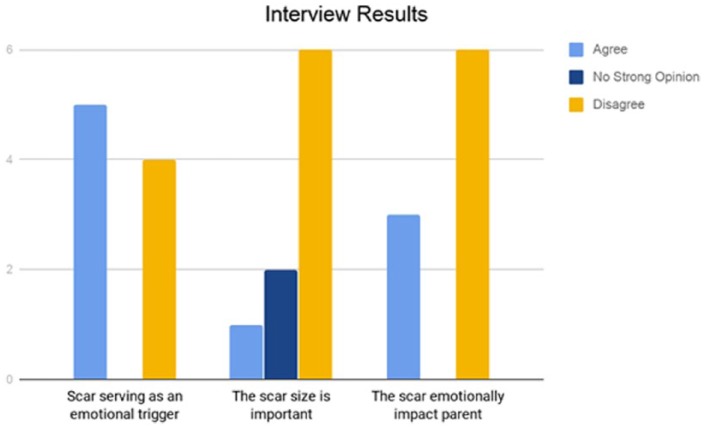
Summary of results from parent interviews.

### Quantitative results

According to the Stony Brook Scar Evaluation Scale, the best possible score is 5, which illustrates a good-looking and well-healed scar. The mean score of photographs of scars received by researches is 4.3, indicating scars were, on average, relatively well healed and insignificant.

## Discussion

### Summary

This was both a qualitative and quantitative study. It aimed to (1) ascertain the severity of scarring from the pigtail chest drain inserted via the Seldinger technique, as measured by the Stony Brook Scar Evaluation Scale, and (2) to understand the emotional impact of these scars on parents through telephone interviews.

It was found that the pigtail chest drain, inserted via the Seldinger technique, did not produce a significant level of scarring. It was also found that while some mothers have a strong negative emotional reaction to the scar, on the whole, the size of scars was not found to be of significant importance to mothers.

### Strengths and limitations of the study

#### Limitations

(1) The study has been conducted using the Stony Brook Scar Evaluation Scale as a method of quantifying the scars. This scale has relatively few criteria and is therefore unable to identify more subtle details and differences that could potentially have been evaluated. (2) Scars were quantified based on photos supplied by mothers. While generally of good quality, it is possible that details of the scar were not adequately represented by a digital image and would have been better measured by an in-person examination. (3) The Stony Brook Scar Evaluation Scale does not distinguish between recent and more aged scars, thereby ignoring the fact that scars may change over time. (4) The small sample size weakens the study’s reliability and quality. Further studies are therefore required to ratify these results.

#### Strengths

(1) Results were generally consistent showing that quantitatively, most scars are small and well healed in nature. Qualitatively, most mothers agreed that the size of the scar is unimportant. This strong sense of agreement among parents in this area shows reliability in results. (2) Data were collected by telephone, as opposed to by written questionnaire, thereby allowing interviewers to further explore themes and encourage more detailed answers.

### Future implications

The results of the research show chest drain scarring size is, overall, relatively unimportant for parents; 3 out of 9 mothers reported that scarring produces a negative emotional response; however, most parents reported that scar size would be irrelevant to this.

From an emotional standpoint, the ideal chest drain would therefore produce no scarring whatsoever. Where scarring is unavoidable however, these results imply that when a NICU is choosing between different types of chest drain, the choice should be guided by chest drain efficacy, rather than its level of scarring, as the emotional impact of the size of chest drain scarring has been shown to be largely unimportant to parents.

Despite these results, it is possible that larger scars may elicit a stronger emotional response from parents. Further studies are needed to investigate this; however, practitioners may want to bear this in mind when choosing between different types of chest drains

A recent 2014 paper compared neonatal pigtail chest drains, inserted via the Seldinger technique, to outcomes achieved from traditional chest tubes, when used to treat pneumothoraces.^7^ Researchers found that while there was no significant difference in safety between drains, the Seldinger-pigtail technique was quicker and easier than the alternative.^7^ While additional research is needed to replicate this article’s findings, evidence suggests that the pigtail drain, as inserted via the Seldinger technique, is a good option for clinicians.
